# LPS and TNF alpha modulate AMPA/NMDA receptor subunit expression and induce PGE2 and glutamate release in preterm fetal ovine mixed glial cultures

**DOI:** 10.1186/1742-2094-10-153

**Published:** 2013-12-17

**Authors:** Luke Weaver-Mikaere, Alistair J Gunn, Murray D Mitchell, Laura Bennet, Mhoyra Fraser

**Affiliations:** 1 The Liggins Institute, The University of Auckland, 85 Park Rd, Grafton, Private Bag 92019, Auckland 1142, New Zealand; 2 Department of Physiology, The University of Auckland, Auckland, New Zealand; 3 University of Queensland Centre for Clinical Research, University of Queensland, Herston, QLD, Australia

**Keywords:** Fetal Sheep, Inflammation, Pre-oligodendrocyte, AMPA, NMDA, NBQX, MK801, COX-2, *in vitro*

## Abstract

**Background:**

White matter injury (WMI) is the major antecedent of cerebral palsy in premature infants, and is often associated with maternal infection and the fetal inflammatory response. The current study explores the therapeutic potential of glutamate receptor blockade or cyclooxygenase-2 (COX-2) inhibition for inflammatory WMI.

**Methods:**

Using fetal ovine derived mixed glia cultures exposed to tumour necrosis factor-α (TNF-α) or lipopolysaccharide (LPS), the expression of alpha-amino-3-hydroxy-5-methyl-4-isoxazole-propionate (AMPA) and N-methyl D-aspartate (NMDA) glutamate receptors and their contribution to inflammation mediated pre-oligodendrocyte (OL) death was evaluated. The functional significance of TNF-α and COX-2 signalling in glutamate release in association with TNF-α and LPS exposure was also assessed.

**Results:**

AMPA and NMDA receptors were expressed in primary mixed glial cultures on developing OLs, the main cell-type present in fetal white matter at a period of high risk for WMI. We show that glutamate receptor expression and configuration are regulated by TNF-α and LPS exposure, but AMPA and NMDA blockade, either alone or in combination, did not reduce pre-OL death. Furthermore, we demonstrate that glutamate and prostaglandin E2 (PGE2) release following TNF-α or LPS are mediated by a TNF-α-COX-2 dependent mechanism.

**Conclusions:**

Overall, these findings suggest that glial-localised glutamate receptors likely play a limited role in OL demise associated with chronic inflammation, but supports the COX-2 pathway as a potential therapeutic target for infection/inflammatory-mediated WMI.

## Background

Maternal infection or infection of the placenta is recognised as one of the most important causes of preterm birth, and of cerebral white matter injury (WMI) in the prematurely born baby [1-4]. Consistent with this, histological chorioamnionitis or raised fetal proinflammatory cytokine levels, including tumour necrosis factor-α (TNF-α), are present in the majority of preterm births [2,5]. At present, the specific mechanisms are incompletely understood, and no effective treatment is available.

Animal models of hypoxic-ischemic WMI have demonstrated that activation of microglia, disturbances in pro-inflammatory cytokine production and release of glutamate are associated with injury via receptor-mediated excitotoxicity [6-8]. In white matter, glutamate release can occur from axons, astrocytes, microglia or from oligodendrocytes (OLs), through the reversal of glutamate transporters [9-15]. OL cell death has been correlated with the expression of Ca^2+^-permeable GluR4-subunit containing alpha-amino-3-hydroxy-5-methyl-4-isoxazole-propionate (AMPA) receptors on developing OLs [7,16,17] and blockade attenuates AMPA-mediated cell death [18]. In contrast, because GluR2 regulates the Ca^2+^-permeability of the AMPA receptor, greater GluR2 expression could be protective against glutamate excitotoxicity.

The developing ovine brain at day 90 of gestation shares a similar susceptibility to WMI to the 24- to 32-week human brain - a period when subcortical white matter is populated predominantly by pre-myelinating OLs [19]. Immature OLs are known to express both AMPA and N-methyl D-aspartate (NMDA)-type glutamate receptors [16,20]. In white matter, AMPA-GluR2 subunit mRNA expression is low throughout gestation [21], thus promoting a Ca^2+^-permeable phenotype, and there is an overall increase in Ca^2+^-permeable AMPA-GluR subunits in pre-OLs compared to mature OLs correlating with greater vulnerability, both *in vitro*[22,23] and *in vivo*[18,24].

Although excitotoxic injury after oxygen-glucose deprivation is well documented [16,25], it is still unknown whether glutamate reaches toxic levels in the intact immature brain exposed to infection. Inflammation activates cyclooxygenase-2 (COX-2) activity [26-28], and there is evidence that prostaglandin E2 (PGE2) stimulates glutamate release from astrocytes [29]. This suggested the hypothesis that inflammation-mediated COX-2 activation would promote release of glutamate in white matter.

The purpose of this study was therefore to examine the contribution of glutamate and its receptor-mediated toxicity to the selective loss of immature pre-OLs in TNF-α or lipopolysaccharide (LPS) induced injury in cultures of preterm fetal ovine primary mixed glia. First, we evaluated glial expression of AMPA and NMDA receptors and determined whether expression levels were responsive to TNF-α or LPS exposure leading to an unfavourable glutamate receptor phenotype. To establish whether this injury involved glutamate receptor activation, we confirmed the presence of AMPA and NMDA receptors on pre-OLs and assessed their role by treating with AMPA or NMDA antagonists, either alone or in combination. We further tested the potential for glia to release glutamate following TNF-α or LPS treatments and whether this was via a TNF-α-COX-2 prostaglandin-mediated mechanism.

## Methods

### Primary mixed glial cultures

Glial cells were isolated from fetal (day 90; term gestation = 145 days) ovine forebrains, characterised by immunocytochemistry detection of cell-specific markers, and maintained in a chemically defined medium, as previously described [30]. Briefly, cerebral cortices with meninges removed were extracted and mechanically dissociated in minimal essential media then digested in Hank’s Balanced Salt Solution (HBSS; Invitrogen Life Technologies, Auckland, NZ) containing trypsin and DNase I and incubated for 60 min at 37°C. The cell suspension was strained through 100 μm cell strainers and separated by a 10% percoll gradient. Cells were washed and resuspended in Dulbecco’s Modified Eagle Medium (DMEM)/F12; Invitrogen Life Technologies) containing 20% horse serum and penicillin and then plated on poly-L-lysine coated 6- and 24-well plates. Glia were initially maintained in DMEM/F12 supplemented with 10% horse serum for 5 days then changed to serum-free DMEM/F12 medium for 24 h before treatment (vehicle, inflammatory agents, inhibitors or antibody). In untreated control cultures O4-oligodendrocytes comprised approximately 7% of total cells in culture while lectin-positive microglia and GFAP-positive astrocytes made up approximately 15% and 78% of total cells, respectively.

### Drug treatment of mixed glial cultures

Prior to treatment with TNF-α (100 ng/mL, Protein Express Inc., Cincinnati, OH, USA) or LPS (1 μg/mL, 055:B5; Sigma-Aldrich) cultures were pre-incubated (30 min at 37°C) with one of five treatments: the AMPA antagonist, NBQX (2,3-dihydroxy-6-nitro-7-sulfamoyl-benzo(*F*)quinoxaline) which blocks AMPA receptors and to a minor degree kainic acid (KA) receptors [31,32] (20 μM in water and media, Sigma-Aldrich Pty. Ltd., Sydney, Australia);MK-801, ((5R,10S)-(+)-5-methyl-10,11-dihydro-5H-dibenzo[a,d]cyclo-hepten-5,10-imine), a highly selective non-competitive antagonist [33] (10 μM in water and media, Sigma-Aldrich Pty. Ltd); NBQX and MK801; the COX-2 inhibitor, NS398 (N-[2-(cyclohexyloxy)-4-nitrophenyl]-methane sulfonamide) [34,35] Cayman Chemical Co., Ann Arbor, MI, USA); 10 μM in 0.013% DMSO and media); or anti-bovine-TNF-α antibody (1:200 in media, Serotec, Oxford, UK). The concentration chosen for each inhibitor was based on previous published studies of cell cultures demonstrating effective inhibition of AMPA and NMDA glutamate receptors [36-38] and COX-2 [39]. Cultures were chronically exposed to TNF-α or LPS alone or in combination with the inhibitors, or the inhibitors alone, for 4 days and assessed at 24-h intervals. Treatments were performed in triplicate across four independent experiments.

### Immunocytochemistry

Following treatment, cells were fixed in 4% paraformaldehyde, rinsed with phosphate-buffered saline (PBS), and incubated with primary antibodies overnight at 4°C. GluR2, GluR4 (Millipore, Temecula, CA, USA; 1:200, 1:500) or NR1 (Sigma-Aldrich Pty. Ltd; 1:200) subunits were localised to O4-positive pre-OLs (Chemicon Int. Inc., CA, USA; 1:100). Secondary Alexa488 conjugates (Invitrogen Life Technologies; 1:800) were incubated for 3 h at room temperature, washed and Hoechst stained before being visualised in TNE buffer. Hoechst 33342 was used as a fluorescent nucleic marker; pre-OL death evaluated by co-localisation of condensed and fragmented nuclei with O4 on immunohistochemistry. Cells were imaged with an inverted fluorescent microscope and composite images generated with Adobe Photoshop (Adobe Systems Incorporated, San Jose, CA). Quantification was performed by counting positively stained cells on four random fields of view, over two wells per experimental replicate.

### Western blotting

Protein lysates were prepared using sodium dodecyl sulfate (SDS) lysis buffer and resuspended in lithium dodecyl sulfate (LDS) sample-buffer (Invitrogen Life Technologies). Samples were electrophoretically separated on 4% to 12% bis-tris precast gels (Invitrogen Life Technologies) and protein bands were electrically transferred to polyvinylidene difluoride membranes (Amersham Biosciences, GE Healthcare, Rydalmere, NSW, Australia). Membranes were blocked for 1 h at room temperature in Tris-buffered saline-Tween (TBS-T; 20 mmol/L Tris–HCl pH 7.6, 132.5 mmol/L NaCl, 0.05% (vol/vol) Tween-20) containing 5% (w/v) non-fat milk powder, then incubated overnight at 4°C with antibodies against GluR2 (Millipore; 1:1,000), GluR4 (Millipore; 1:100) or NR1 (Sigma-Aldrich Pty. Ltd., Sydney, Australia; 1:2,000) in TBS-T supplemented with 5% BSA. Membranes were incubated for 2 h at room temperature with horseradish peroxidase-conjugated anti-rabbit or anti-mouse antibodies (Thermo Scientific, Rockford, IL, USA; 1:2,000) in TBS-T supplemented with 5% non-fat milk powder. Bands were visualised on X-ray films using ECL-plus substrate (Thermo Scientific) as per the suppliers’ instructions. Between all incubation steps, membranes were washed extensively with TBS-T.

### Quantitative real-time PCR

Total RNA was isolated by washing cells in ice-cold PBS, followed by suspension in TRIzol (Invitrogen, Rockford, IL, USA) and then stored at -80°C. mRNA was extracted using the RNeasy Mini Kit (Qiagen, Valencia, CA, USA), according to the manufacturer’s instructions. Total RNA (final concentration 5 to 10 ng/μL) was incubated with random primers and deoxynucleotide mix and incubated at 65°C for 7 min then reverse-transcribed in superscript III (Invitrogen Life Technologies) according to the manufacturer’s protocol. GluR1-4 and NR1 transcript abundance was determined by real-time PCR using TaqMan Gene Expression Assays (Applied Biosystems, Foster City, CA, USA). Amplification of GluR1, R2, R3 R4 and NR1 gene transcripts were performed in triplicate on an ABI PRISM 7900HT Sequence Detector (Applied Biosystems) using standard cycling conditions recommended by the manufacturer (Melt; 15 s, 95°C, anneal/extend; 1 min 60°C for 40 cycles). Singleplex amplification was performed with a total reaction volume of 10 μL, containing 5 μL TaqMan Universal PCR Master Mix (Applied Biosystems), 1 μL cDNA template, 250 nM probe, 900 nM forward and reverse primers and 2.75 μL diethylpyrocarbonate (DEPC)-treated water. Standard curves of the target gene and the house-keeping gene, 18S, were included in each plate, consisting of five-fold serial dilutions of cDNA synthesised from an LPS-treated fetal ovine cerebellum. Primer and probes sets for all target genes were designed using the Primer Express software (Applied Biosystems). Analysis was performed using the relative standard curve method and expressed as fold change expression relative to time-matched controls. GluR2 was also expressed relative to time-matched controls, normalised to the expression of all other AMPA subunits.

### Glutamate measurement

Glutamate concentrations in media before and after treatment with TNF-α or LPS and with or without NS398 or the TNF-α antibody, were determined using the Amplex®Red glutamic acid/glutamate oxidase assay kit (Invitrogen Life Technologies) according to the manufacturer’s instructions. Briefly, the reaction buffer (consisting of Amplex red reagent, L-alanine, glutamic acid oxidase and glutamate-pyruvate transaminase) was added to samples of culture media and glutamate standards and incubated in the dark for 30 min at 37°C. Fluorescence was measured at an excitation wavelength of 544 nm and an emission wavelength of 590 nm with a multi-mode plate reader (BioTek Synergy 2, Winooski, VT, USA). Relative fluorescent units (RFU) were converted based on the RFU measurement of the known standard concentrations and adjusted for total protein content in the media sample (determined by bicinchoninic acid (BCA) assay; Thermo Scientific). Samples were assessed in duplicate and the final concentration represented as mM of glutamate per mg of total protein.

### Prostaglandin E2 immunoassay

PGE2 concentrations in media were determined by direct radioimmunoassay, similar to that described previously [40]. Conditioned media samples (1:50) and standards (0–5,000 pg/mL, Caymen Chemical Co.) were dissolved in serum-free DMEM/F-12 medium and incubated in PGE2 tritiated tracer (approximately 5,000 cpm/100 μL; Perkin Elmer, Waltham, MA, USA) and antisera (raised in-house in rabbits against PGE2-BSA and PGE2-thyroglobulin conjugates) overnight at 4°C. Unbound PGE2 and tracer were removed with cold dextran-coated charcoal, the supernatant mixed with scintillation fluid (Ultima Gold, Perkin Elmer) and counted with a Tri-Carb 2910TR Liquid Scintillation Analyzer (PerkinElmer, Inc., Downers Grove, IL, USA). Curve fitting and data extrapolation were performed using QuantaSmart software (PerkinElmer, Inc.). Media containing TNF-α or LPS, with and without the COX-2 inhibitor, were included as no treatment controls. The sensitivity of the PGE2 radioimmunoassay ranged from 1 to 7 pg/mL, with an intra-assay precision of 5.6% and an interassay precision of 17.8%.

### Statistical analysis

Data are presented as mean ± standard error of the mean (SEM). Statistical comparisons between groups were performed using two-way analysis of variance (ANOVA). Where significant differences were observed, Tukey’s post-hoc analysis was used for comparisons of the means unless otherwise stated. The significance level was set at *P* <0.05.

## Results

### Oligodendrocyte survival is not effectively preserved by glutamate receptor inhibition in activated mixed glial cultures

**Figure 1:**
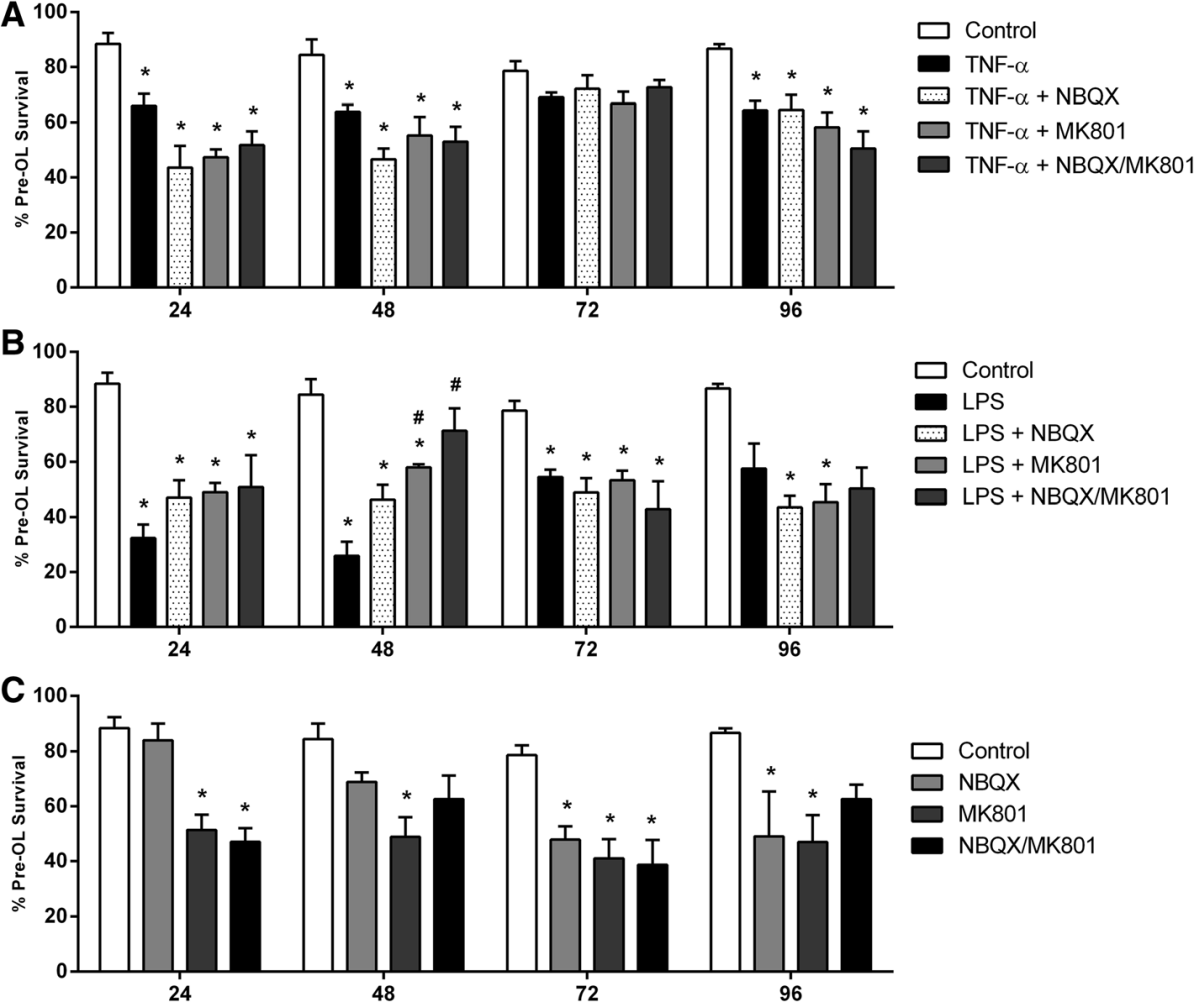
**Pre-OL survival in activated mixed glial cultures exposed to AMPA or NMDA receptor antagonists.** Immunocytochemistry was used to assess pre-OL survival in mixed glial cultures exposed to TNF-α **(A)** or LPS **(B)** alone or without **(C)** or in combination with NBQX, MK-801, or combined NBQX/MK-801. Data are presented as mean pre-OL survival as a percentage of total pre-OL + SEM of four independent experiments. * indicate a significant difference (*P* <0.05) between treated means compared to time-matched control and # indicate a significant difference (*P* <0.05) compared to TNF-α or LPS treated means.

TNF-α significantly reduced pre-OL survival after 24, 48 and 96 h (*P* <0.05, *P* <0.01, *P* <0.05; Figure 1A) while LPS induced a marked reduction in survival from 24 to 72 h (*P* <0.001, *P* <0.001, *P* <0.05; Figure 1B). NBQX, an antagonist of the AMPA-kainate subtype, and MK-801, an antagonist of the NMDA subtype of glutamate receptors, did not increase pre-OL survival either separately or in combination following TNF-α treatment. However, in LPS-treated cultures at 48 h, NMDA inhibition transiently improved pre-OL survival compared to LPS treatment alone, and combined AMPA/NMDA inhibition improved pre-OL survival to a level comparable to untreated cells (*P* <0.001; Figure 1B).

Pre-OL survival was also evaluated in cultures treated with inhibitors alone to assess potential toxic effects of glutamate receptor inhibition (Figure 1C). NBQX alone was associated with a gradual reduction of pre-OL survival with significant reductions seen after 72 and 96 h of treatment (*P* <0.05), while MK-801 was associated with a significant decline in pre-OL survival at all time-points assessed (*P* <0.05). Combined NBQX/MK-801 substantially reduced pre-OL survival at 24 and 72 h (*P* <0.05), however despite this decrease, no significant change in survival compared to controls was observed at 48 and 96 h.

These results suggest that AMPA and NMDA receptor activation may not be primary contributors to inflammation-induced pre-OL injury and raise the possibility that a lack of improved survival after exposure to NBQX and/or MK-801 may be due in part at least to a general adverse effect on survival in cultures of mixed glia.

### TNF-α and LPS alter GluR2 subunit expression

**Figure 2:**
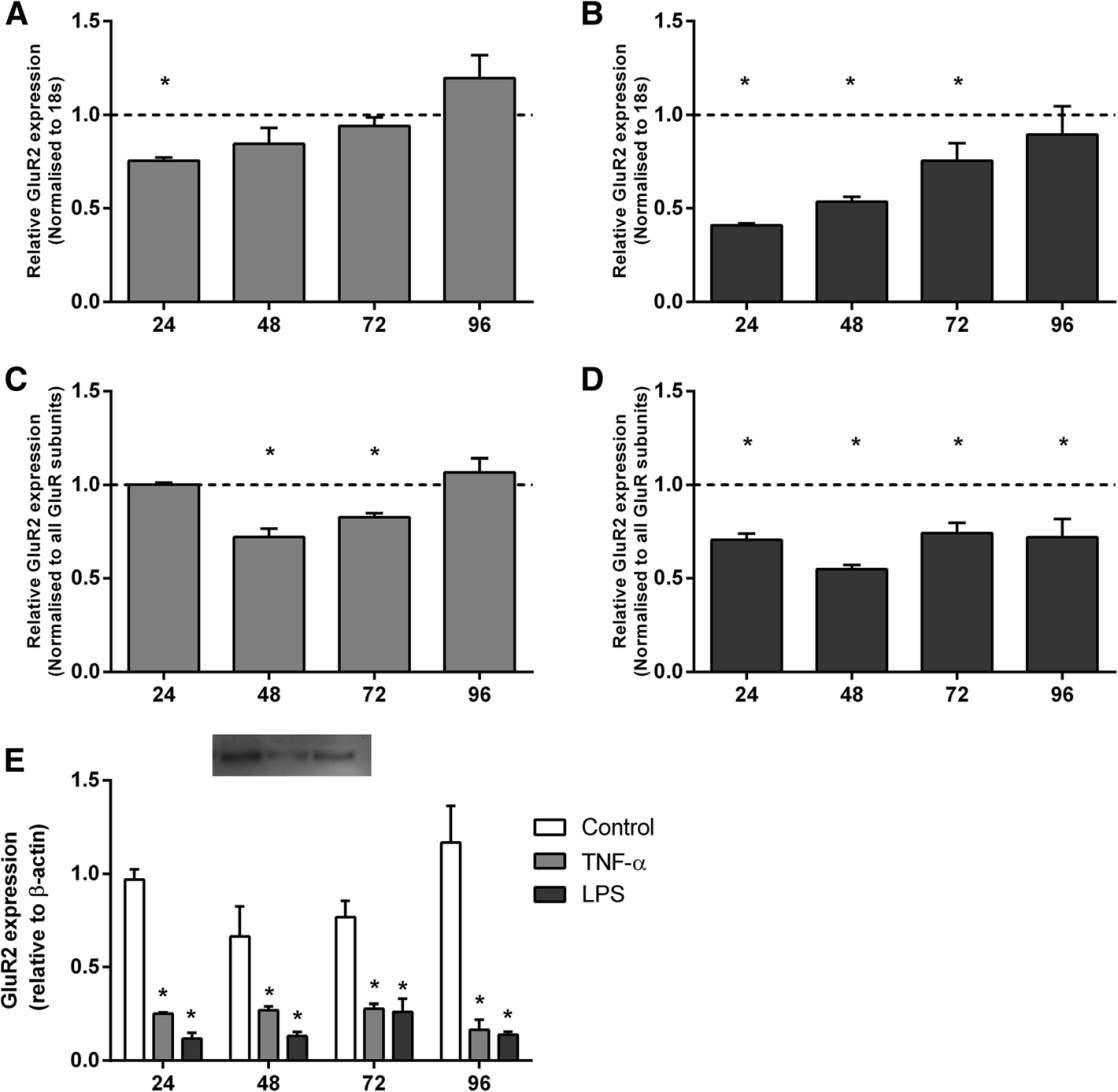
**GluR2 mRNA and protein expression in activated mixed glial cultures.** Quantitative real-time PCR **(A-D)** and western blotting **(E)** were performed to detect the AMPA subunit, GluR2, expressed in mixed glial cultures in the absence and presence of TNF-α **(A, C)** or LPS **(B, D)**. Data are presented as fold change in gene expression relative to time-matched controls and standardised to 18 s or to all other GluR subunits. Protein expression is relative to β-actin + SEM of four independent experiments. * *P* <0.05.

**Figure 3:**
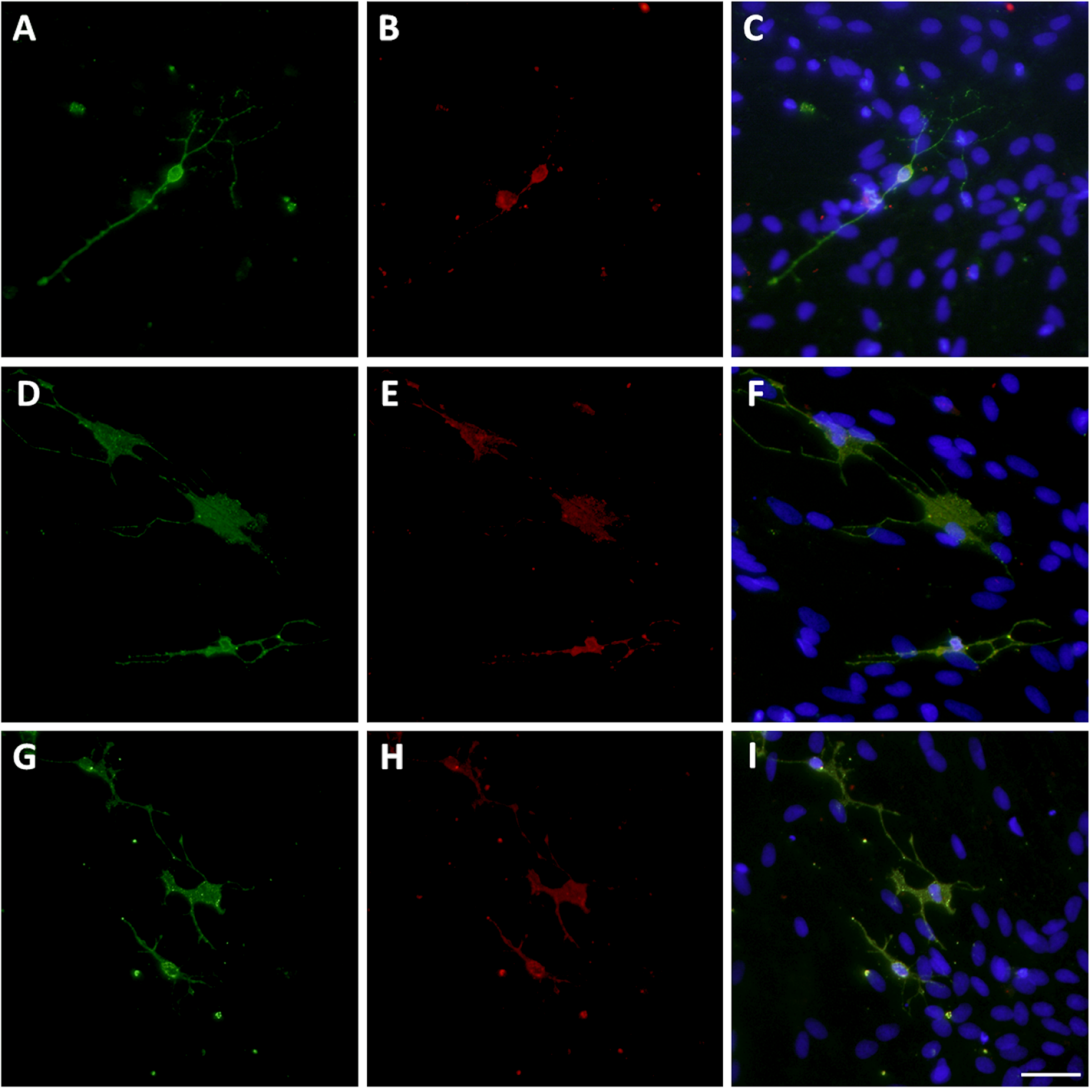
**GluR2 expression on pre-OLs.** Untreated controls **(A-C)** or cultures chronically incubated with TNF-α **(D-F)** or LPS **(G-I)** were assessed immunocytochemically for GluR2 subunit expression (red) co-localised with pre-OLs (green). The nucleus was stained with Hoechst fluorescent dye (20 × magnification), scale bar = 10 μm.

GluR2 mRNA expression was significantly reduced at 24 h (*P* <0.05; Figure 2A) following TNF-α exposure, and was significantly reduced during 24 to 72 h of LPS exposure (*P* <0.05; Figure 2B). There was a marked decline in GluR2 protein expression at all time-points following TNF-α and LPS exposure as determined by western blot (Figure 2E). Immunocytochemistry showed modest numbers of pre-OLs in untreated and TNF-α and LPS treated cultures co-localised with GluR2 (Figure 3).

Importantly, it is the relative expression of the GluR2 subunit to the other AMPA subunits that regulates Ca^2+^-permeability. Analysis of GluR2 expression normalised to the expression of all other AMPA subunits (GluR1, GluR3 and GluR4) revealed a significant reduction at 48 and 72 h following TNF-α exposure (*P* <0.05; Figure 2C), and was significantly reduced at all time-points following LPS exposure (*P* <0.05; Figure 2D).

### TNF-α and LPS alter GluR4 subunit expression

**Figure 4:**
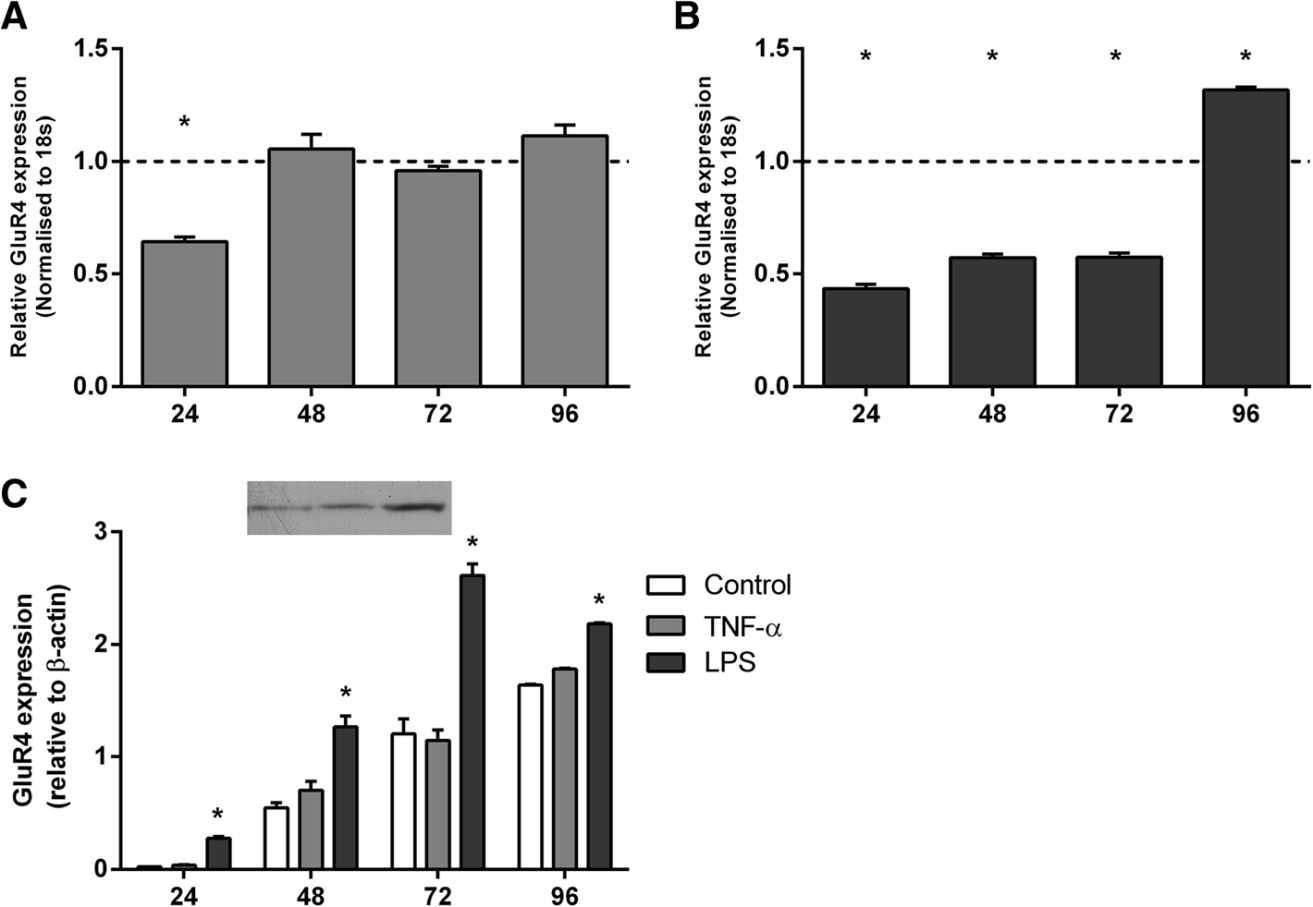
**GluR4 mRNA and protein expression in activated mixed glial cultures.** Quantitative real-time PCR **(A, B)** and western blotting **(C)** were performed to detect the AMPA subunit, GluR4, expressed in mixed glial cultures in the absence and presence of TNF-α **(A)** or LPS **(B)**. Data are presented as fold change in gene expression relative to time-matched controls and standardised to 18 s. Protein expression is relative to β-actin + SEM of four independent experiments. * *P* <0.05.

TNF-α induced a transient reduction of GluR4 mRNA expression after 24 h (*P* <0.05; Figure 4A) while LPS treatment resulted in a marked sustained reduction from 24 to 72 h (*P* <0.05; Figure 4B) and a delayed increase after 96 h (*P* <0.05).

LPS exposure resulted in a robust increase in GluR4 protein expression at all time-points (*P* <0.05; Figure 4C) as determined by western blot.

**Figure 5:**
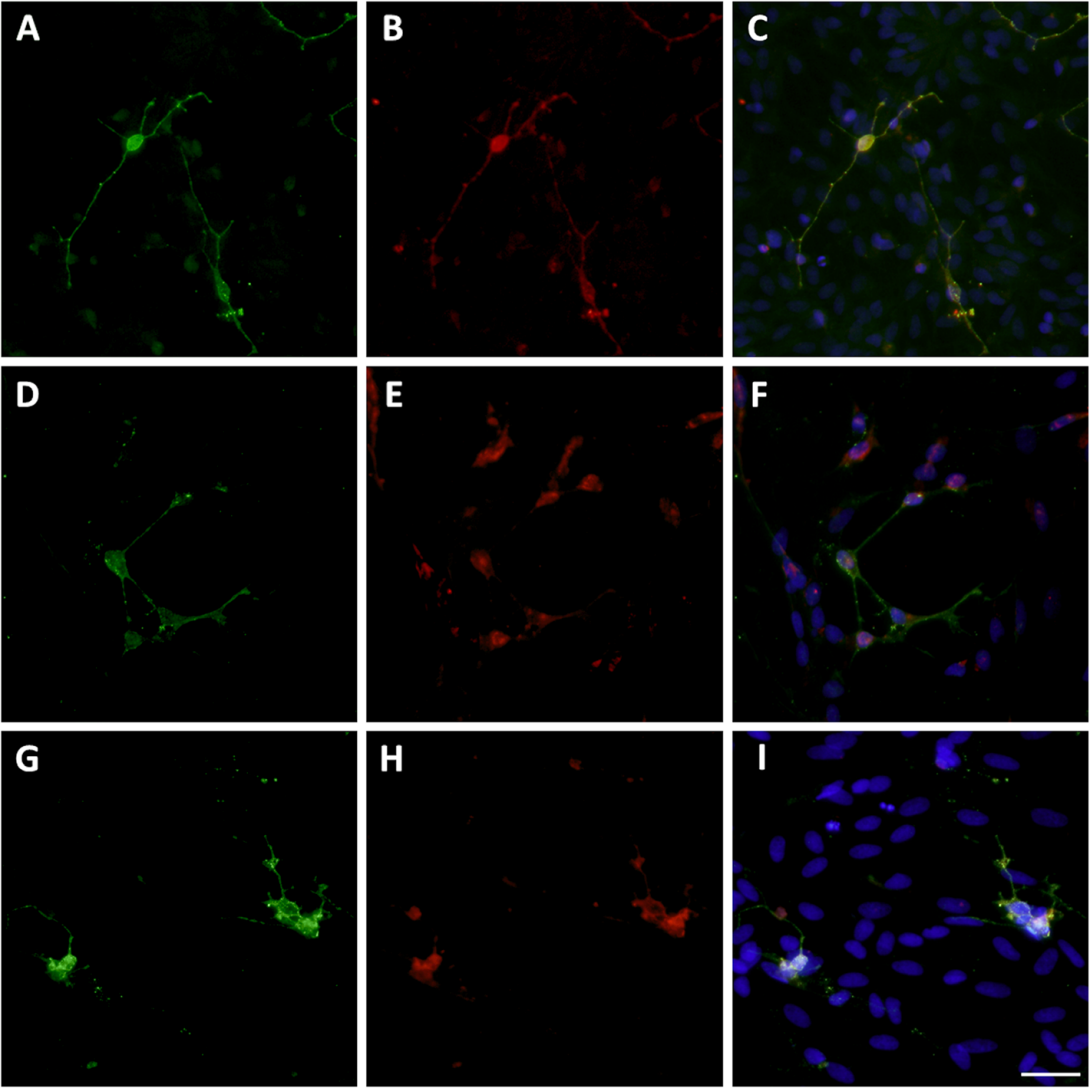
**GluR4 expression on pre-OLs.** Untreated controls **(A-C)** or cultures chronically incubated with TNF-α **(D-F)** or LPS **(G-I)** were assessed immunocytochemically for GluR4 subunit expression (red) co-localised with pre-OLs (green). The nucleus was stained with Hoechst fluorescent dye (20 × magnification), scale bar = 10 μm.

In untreated control cultures, GluR4 immunoreactivity was strong at the cell body with patches of staining on pre-OL processes, and pre-OLs in immune stimulated cultures also exhibited a strong immunoreactivity to GluR4 (Figure 5).

### TNF-α and LPS alter NMDA glutamate receptors

**Figure 6:**
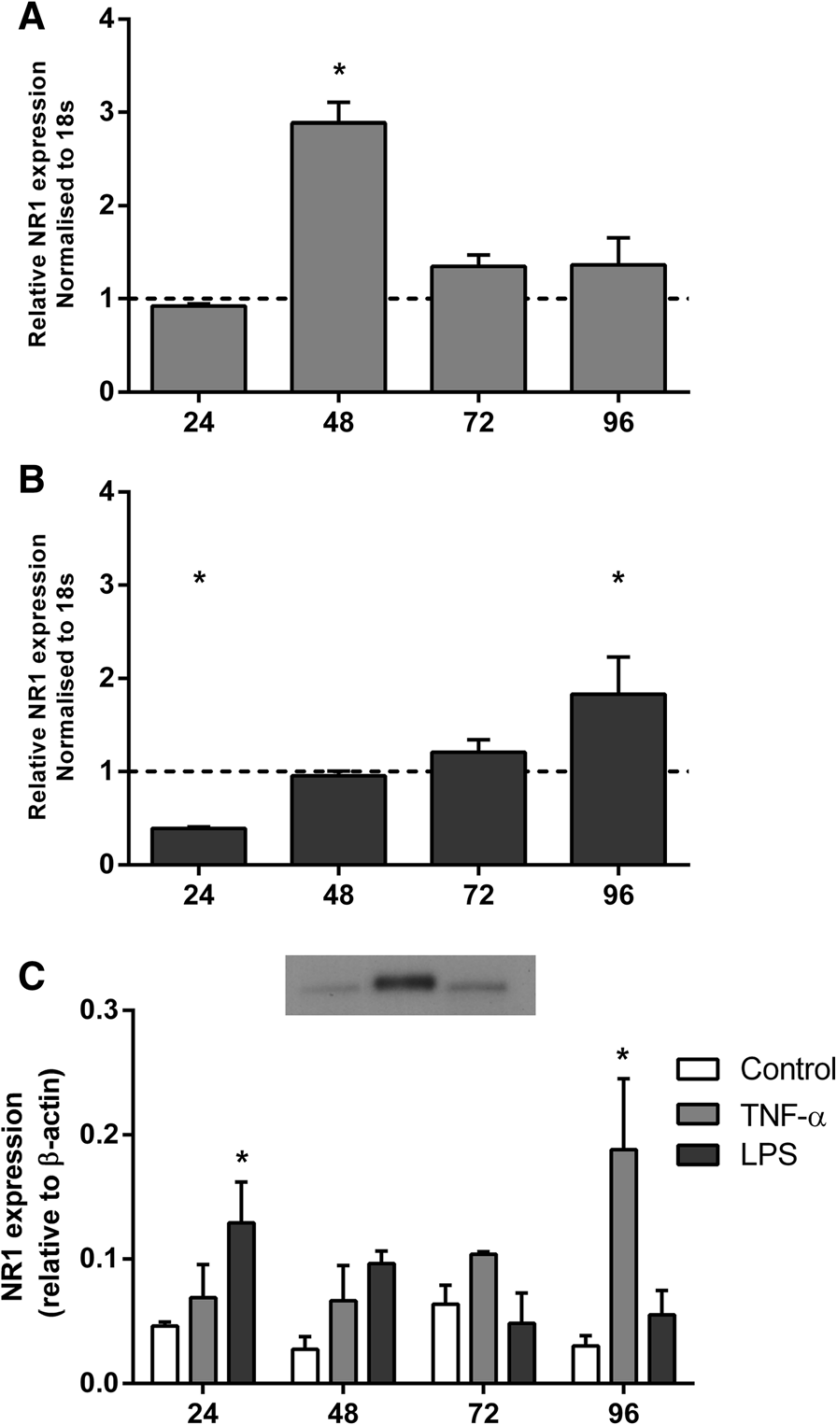
**NR1 mRNA and protein expression in activated mixed glial cultures.** Quantitative real-time PCR **(A and B)** and western blotting **(C)** were performed to detect the NMDA subunit NR1 and AMPA subunit GluR2 expressed in mixed glial cultures in the absence and presence of TNF-α or LPS. Data are presented as fold change in gene expression relative to time-matched controls and standardised to 18 s + SEM of four independent experiments. * *P* <0.05.

A marked transient increase in mRNA expression of the obligatory NMDA subunit, NR1, was induced by TNF-α after 48 h (*P* <0.05; Figure 6A), while LPS treatment resulted in a delayed increase after 96 h (*P* <0.05; Figure 6B). Likewise, TNF-α and LPS increased protein expression of NR1 after 24 h (Figure 6C) and 72 h (*P* <0.05), respectively, by western blot.

**Figure 7:**
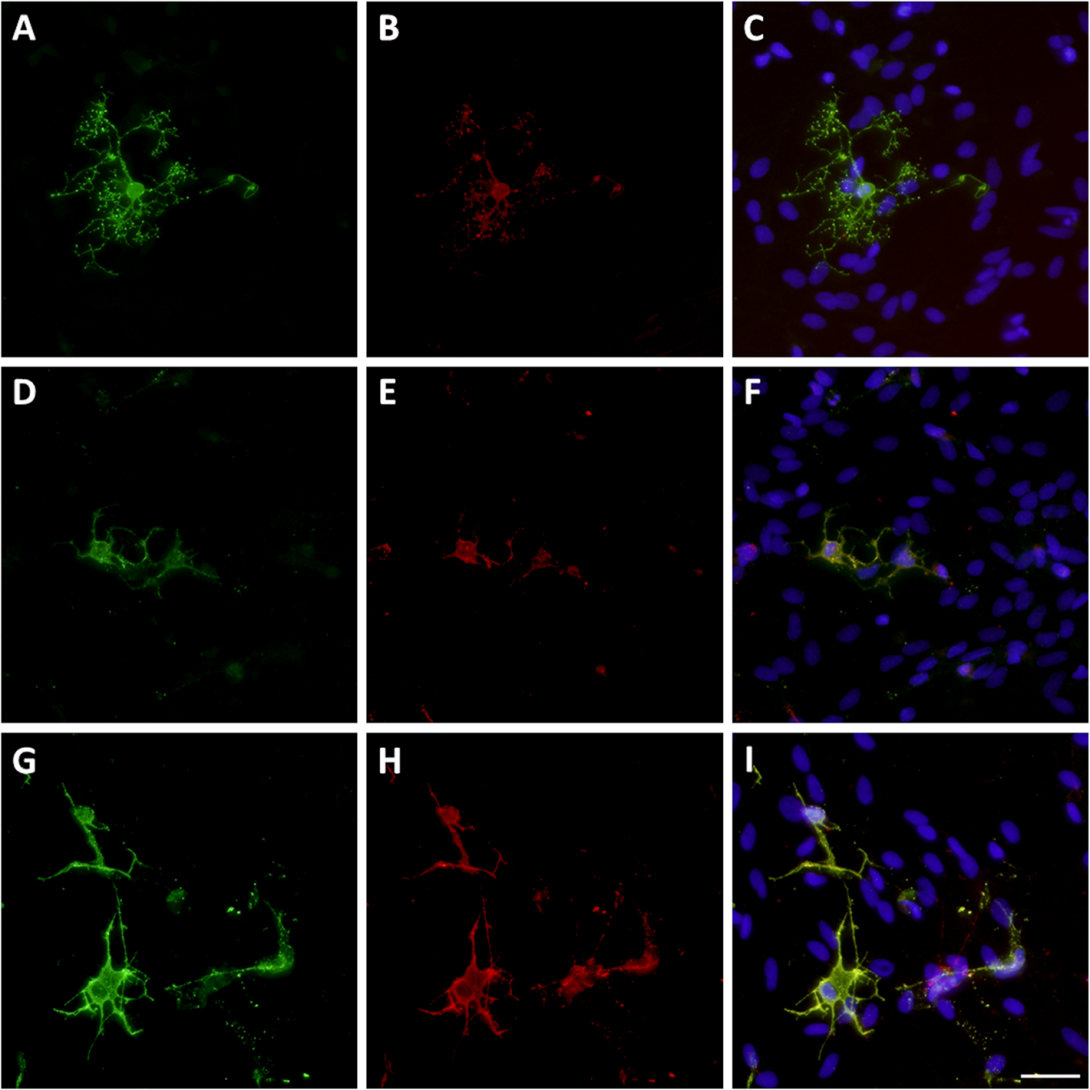
**NR1 expression on pre-OLs.** Untreated controls **(A-C)** or cultures chronically incubated with TNF-α **(D-F)** or LPS **(G-I)** were assessed immunocytochemically for NR1 subunit expression (red) co-localised with pre-OLs (green). The nucleus was stained with Hoechst fluorescent dye (20 × magnification), scale bar = 10 μm.

NR1 staining was seen on pre-OLs, with moderate staining occurring across most of the cell body and processes in untreated cultures. Pre-OLs in immune stimulated cultures exhibited a strong immunoreactivity to the NR1 subunit (Figure 7).

### TNF-α and LPS mediate increased extracellular glutamate levels via a TNF-α and COX-2-dependent mechanism

It has been previously shown that PGE2 stimulates glutamate release from astrocytes [29]. While information is available on the inflammation-mediated release of glutamate from glia [41,42] and the intact adult brain [43,44] there is a paucity of data with respect to the immature brain. To evaluate whether TNF-α and LPS act via PGE2 to influence glutamate release in white matter, PGE2 and glutamate concentrations were assessed in cultures of mixed glia exposed to TNF-α and LPS.

**Figure 8:**
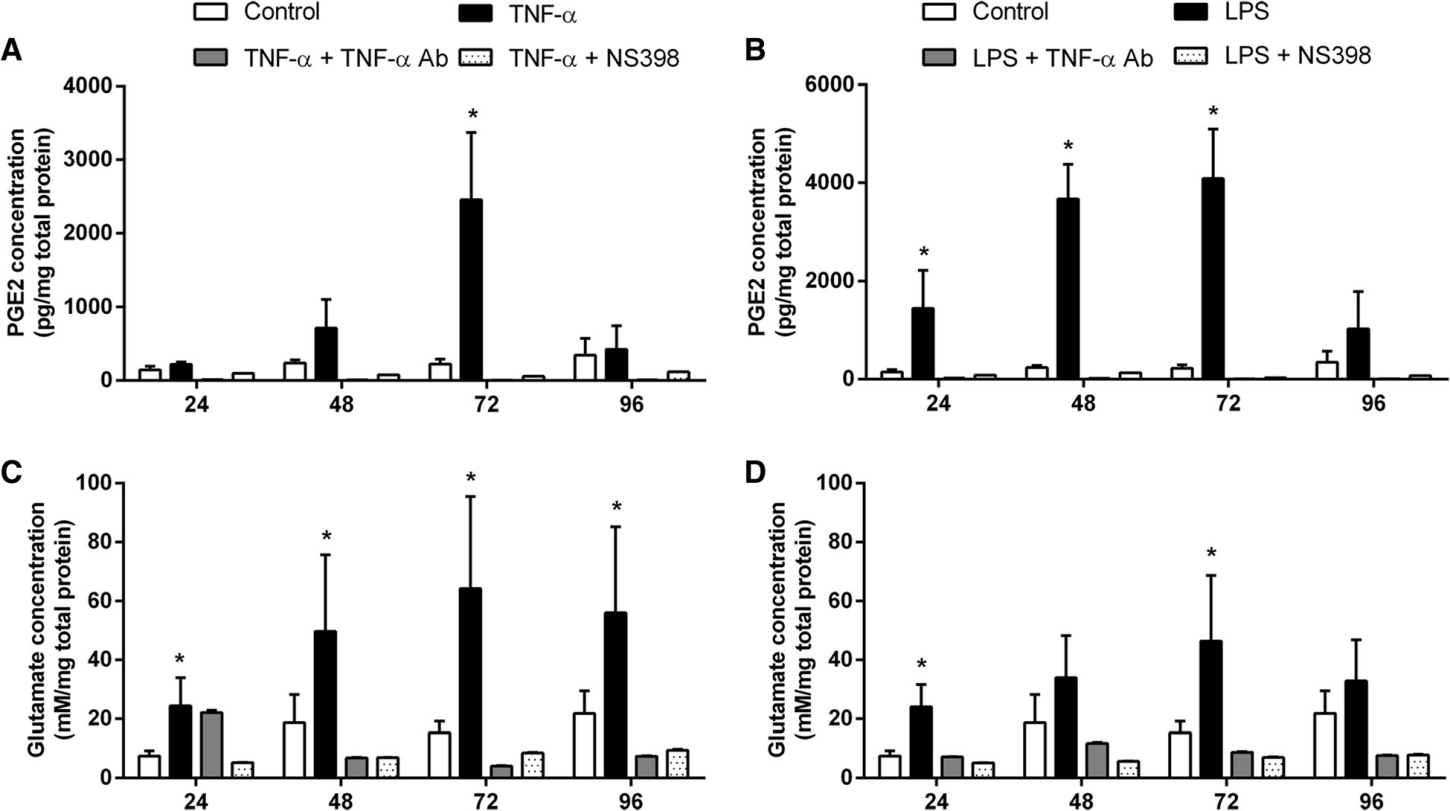
**Prostaglandin E2 (PGE2) and glutamate concentrations in conditioned media from activated mixed glial cultures.** Concentrations of secreted PGE2 **(A and B)** or glutamate **(C and D)** were determined in cultures of mixed glial cells in the absence and presence of TNF-α or LPS. Data are presented as mean concentration, adjusted to total protein concentration, + SEM of four independent experiments. * *P* <0.05, compared to controls.

TNF-α exposure resulted in a gradual increase in the PGE2 concentration in culture media, with a significant increase observed only after 72 h of treatment (*P* <0.05; Figure 8A), whereas LPS exposure increased PGE2 concentrations from 24 to 72 h (*P* <0.05; Figure 8B). COX-2 inhibition completely abolished the increase in PGE2 in both treatment groups and TNF-α antibody treatment resulted in a similar reduction indicating that activation of the TNFR1 likely activates COX-2-mediated PGE2 release.

Interestingly, TNF-α significantly increased the amount of glutamate in the culture media at all time-points (*P* <0.05; Figure 8C) whereas glutamate concentrations were only significantly increased at 24 and 72 h following LPS treatment (*P* <0.05; Figure 8D). TNF-α antibody and NS398 treatments substantially inhibited the induced increase in extracellular glutamate compared to TNF-α and LPS treatment alone, resulting in levels comparable to those detected in untreated controls. Hence, stimulation with TNF-α or LPS greatly contributes to glutamate release and appears to be directly dependent on a TNF-α-COX-2 pathway.

### Pre-OL survival is preserved by TNF-α and COX-2 inhibition in activated mixed glial cultures

**Figure 9:**
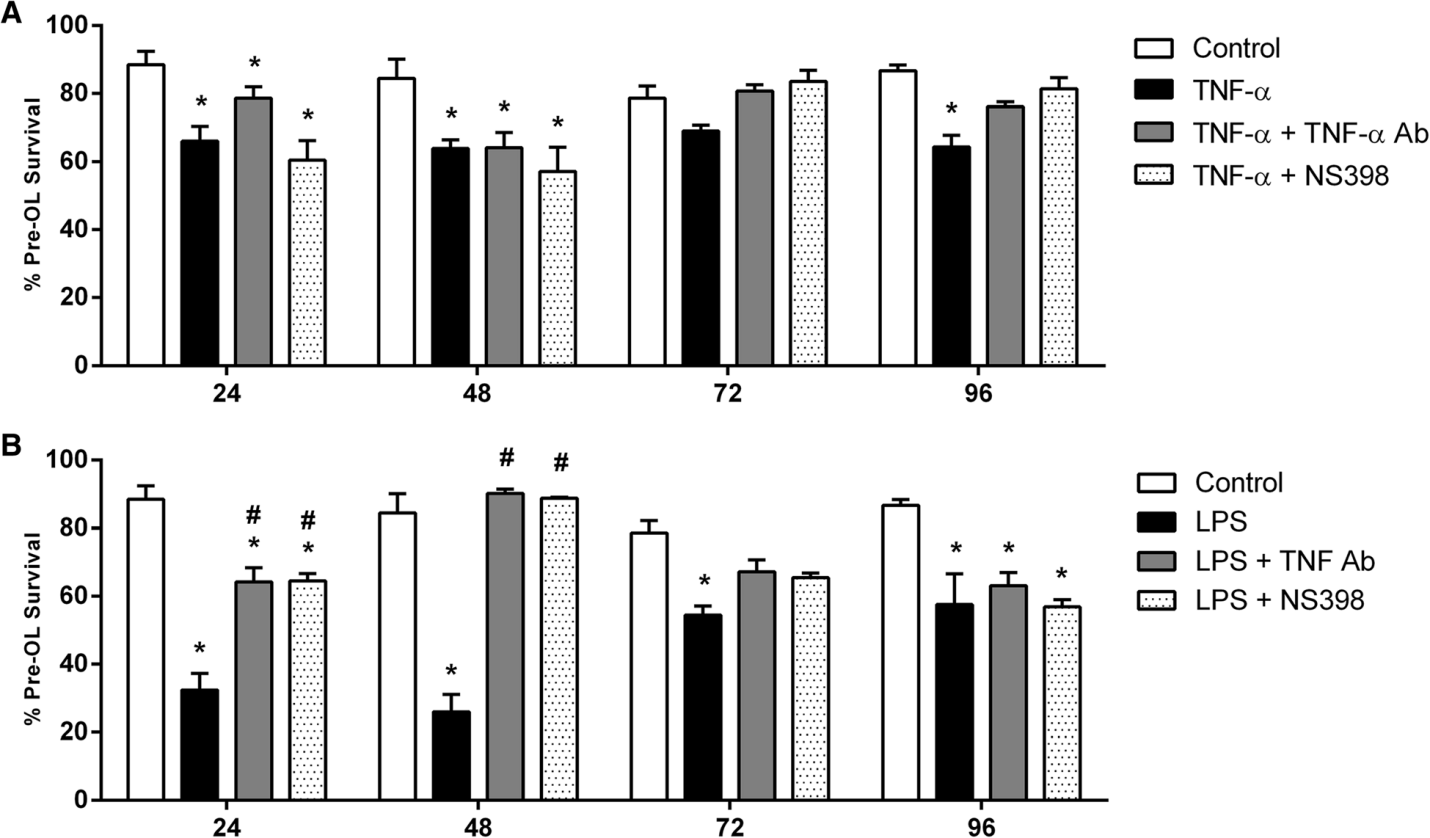
**Pre-OL survival in activated mixed glial cultures exposed after COX-2 and TNF-α inhibition.** Immunocytochemistry was used to assess pre-OL survival in mixed glial cultures exposed to TNF-α **(A)** or LPS **(B)** alone or in combination with the COX-2 inhibitor, NS398, or TNF-α antibody treatment. Data are presented as mean pre-OL survival as a percentage of total pre-OLs + SEM of four independent experiments. * indicate a significant difference (*P* <0.05) between treated means compared to time-matched control and # indicate a significant difference (*P* <0.05) compared to TNF-α or LPS treated means.

We evaluated the consequences of TNF-α and COX-2 blockade to determine if effectors downstream of TNF-α or PGE2 signalling contribute to inflammation-mediated OL death. TNF-α significantly reduced pre-OL survival after 24, 48 and 96 h (*P* <0.05; Figure 9A) and LPS reduced survival from 24 to 72 h (*P* <0.05; Figure 9B), whereas TNF-α induced pre-OL loss was delayed by treatment with a TNF-α antibody until 48 h. However, in combination with LPS, TNF-α antibody treatment significantly improved pre-OL survival at 24 and 48 h compared to LPS treatment alone (*P* <0.05) but did not significantly protect after 96 h of LPS treatment.

NS398 co-treatment in TNF-α exposed cultures resulted in pre-OL levels comparable to those after TNF-α alone in the first 48 h (*P* <0.05). After 72 h, pre-OL survival in TNF-α and NS398 co-treated cultures was comparable to those in untreated controls. In LPS-exposed cultures co-treated with NS398 pre-OL survival was significantly improved at 24 and 48 h compared to LPS treatment alone (*P* <0.05).

Overall, in TNF-α treated cultures, pre-OL survival was maintained at levels of controls at the later time-points with TNF-α antibody and NS398 co-treatments. However, TNF-α antibody and NS398 treatments noticeably improved pre-OL survival in the immediate 48 h after LPS exposure. These results suggest that TNF-α and COX-2 may contribute to inflammation-induced pre-OL injury either directly or via downstream effectors.

## Discussion

There is currently no available treatment for infection-related WMI in preterm infants. The present study confirms the presence of AMPA and NMDA receptors on pre-OLs in cultures of mixed glial cells derived from the preterm ovine fetus at a gestational age when white matter maturity is most similar to preterm human infants [19]. Furthermore, it demonstrates that AMPA/NMDA blockade does not protect against pre-OL injury and that PGE2 release plays a key role in inflammation-mediated increases in glutamate levels and loss of pre-OLs. These findings suggest that TNFα-COX-2-related pathways may be an important therapeutic target for inflammation-induced brain injury.

This is the first observation in ovine-derived fetal glia that GluR2 subunit mRNA and protein expression (both absolute and relative to GluR1, 3 and 4 subunits) are transiently reduced after TNF-α exposure and persistently after LPS. Furthermore, pre-OLs expressed the Ca^2+^-permeable AMPA subunit GluR4, and although mRNA expression was only increased in LPS-treated cultures after 96 h, GluR4 protein was persistently increased. Reduced expression of GluR2 compared to GluR4 (and other GluR subunits) in association with a delayed rise in extracellular glutamate concentrations suggests increased potential for formation of more Ca^2+^-permeable AMPA receptors in ovine glia, including pre-OLs and thus potentially more vulnerable to excitotoxicity [45,46].

We confirmed that ovine pre-OLs express the NR1 subunit in primary mixed glial cultures. NR1 protein expression, however, was differentially expressed by TNF-α and LPS with an acute, transient increase after LPS exposure and a gradual increase by 96 h after TNF-α exposure. Others have shown that NMDA receptors are expressed by OLs and contribute to white matter pathology after hypoxia-ischemia, by triggering loss of processes and increasing apoptosis and generation of reactive species [20,47,48]. We speculate that the initial increase in NR1 expression after LPS may increase loss of pre-OL processes. While these data suggest that pre-OL process-localised NMDA receptors may contribute to acute phase white matter damage in inflammation, further analysis of simultaneous localisation of GluR2 and GluR4 and O4 are needed to confirm this. The differential pre-OL survival after TNF-α compared to LPS may reflect the more complex inflammatory environment induced by LPS. While TNF-α is an inflammatory cytokine, the LPS epitope (Escherichia coli O55:B5) used in the present study is known to robustly modulate both pro- and anti-inflammatory cytokines through toll-like receptor 4 and 2 stimulation [49]. In neonatal-derived glial cultures, LPS stimulates an increase in endogenous levels of the pro-inflammatory cytokine, interleukin-1β (IL-1β), which mediates the inflammatory actions of LPS by NO [50] and an increase in the anti-inflammatory cytokine, IL-10 [51], which can confer protection against oligodendroglial death [52]. Thus, LPS produces a much more intricate gene response than TNF-α alone. Further, astrocytes have key homeostatic and glio-transmission functions and it is unknown whether astrocytic GluR subunit expression is differentially affected by TNF-α or LPS exposure.

Data from the current study show that glial derived extracellular glutamate levels are substantially increased in response to both TNF-α and LPS. To the best of our knowledge this is the first report in mixed glial cultures and is potentially important given that as discussed above, there is the potential for excitotoxic injury via the activation of more Ca^2+^-permeable glutamate receptors. Others suggest the major source of glutamate in white matter lesions is by reversal of sodium glutamate transporters [53] from pre-OLs and axons [11], however, it is also likely that astrocytes, directly, and microglia, indirectly, contribute to this. Indeed, we have previously shown that TNF-α is increased in LPS-exposed preterm fetal ovine mixed glial cultures [30], likely released by microglia that could potentially inhibit the regulatory glutamate uptake by astrocytes [54].

The present studies show that the TNF-α and LPS-induced increase in glutamate concentrations are mediated via a TNF-α and COX-2 dependent mechanism. Our findings are consistent with TNF-α signalling via TNFR1 initiating production of PGE2, and TNF-α and COX-2 inhibition improved pre-OL survival 48 h after LPS exposure. A role for COX-2 has already been established in neuronal LPS-induced injury [39] and COX-2 inhibition limited OL death in encephalomyelitis and an *in vitro* excitotoxic model [55]. Thus, the TNF-α-COX-2 pathway represents a potential therapeutic target for OL protection during neuroinflammation which requires further confirmation using other models of injury such as the chronically catheterised preterm fetal sheep model.

Other cytokines may also activate COX-2. For example, exogenous (IL-1β) induced the release of PGE2 via activation of nuclear factor-kappa B (NFκB) and mitogen-activated protein kinase (MAPK) cascades [56]. However, in neonatal-derived mixed glial cultures endogenous IL-1β was much less potent than LPS in inducing PGE2 production, suggesting that it is not a major mediator [50]. Overall our results suggest that TNF-α and COX-2 may contribute to inflammation-induced pre-OL injury either directly or via downstream effectors.

In contrast, our data show that treatment with NBQX and/or MK-801 following TNF-α or LPS exposure was not protective against pre-OL death, suggesting that AMPA and NMDA receptor activation do not make a major contribution to inflammation-induced pre-OL injury. Consistent with this, in near-term fetal sheep treatment with topiramate, an AMPA receptor antagonist, 5 h after severe cerebral ischemia did not protect white matter or grey matter [57]. Indeed, high doses of topiramate are associated with increased neuronal and white matter apoptosis in the normal developing brain [58,59]. We confirmed that the lack of improved pre-OL survival may be due to toxic effects of NMDA and combined AMPA/NMDA inhibition. Nevertheless, there were no apparent adverse effects of chronic AMPA inhibition alone within the first 48 h. These findings support that TNF-α and LPS-induced loss of pre-OLs at early time-points in our study (24 and 48 h) is likely independent of both glutamate receptor inhibition and any potential toxic effects of NBQX. Furthermore, our findings and previous reports of adverse effects of glutamate blockade [60,61] suggest that modulating glutamate receptor signalling risks modifying brain development and may have deleterious consequences for pre-OLs.

In summary, this study suggests that AMPA and NMDA glutamate receptor inhibition does not seem to be an effective therapy for inflammation-mediated pre-OL death. Glutamate levels and glutamate receptor conformation were altered by inflammatory stimuli, leading to a delayed rise in extracellular glutamate concentrations combined with a receptor phenotype that is favourable to Ca^2+^-permeability over 4 days after exposure. Critically, we observed that the increase in extracellular glutamate concentrations occurred after the onset of loss of pre-OL cells. This strongly suggests that the increase in glutamate may have been a consequence of cell death, perhaps due to impaired energy dependent reuptake or to release from lysing cells, rather than the cause of injury.

We conclude that our hypothesis that glutamate receptor-mediated toxicity contributes to WMI requires refinement to address the overall lack of effect of receptor inhibition. In contrast, the TNF-α-Cox-2 pathway should be further explored as a potentially viable therapeutic target, to protect pre-OLs from neuroinflammatory damage of the immature brain.

## Abbreviations

AMPA: Alpha-amino-3-hydroxy-5-methyl-4-isoxazole-propionate; COX-2: Cyclooxygenase 2; LPS: Lipopolysaccharide; NMDA: N-methyl D-aspartate; OL: Oligodendrocyte; PGE2: Prostaglandin E2; TBS-T: Tris-buffered saline-Tween; TNF-α: Tumour necrosis factor-α; WMI: White matter injury.

## Competing interests

The authors declare that they have no competing interests.

## Authors’ contributions

LW-M, AJG, LB, MM and MF conceptualised the study. LW-M and MF designed the protocol and carried out the glial cultures, and molecular and protein expression; LW-M and MF analysed data. AJG and MF provided critical interpretation. LW-M, AJG, LB, MM and MF drafted the paper. All authors have read and approved the final version of the manuscript.
